# A neural tract tracing study on synaptic connections for cortical glutamatergic terminals and cervical spinal calretinin neurons in rats

**DOI:** 10.3389/fncir.2023.1086873

**Published:** 2023-04-28

**Authors:** Ziyun Huang, Liping Sun, Xuefeng Zheng, Ye Zhang, Yaxi Zhu, Tao Chen, Zhi Chen, Linju Ja, Lisi OuYang, Yaofeng Zhu, Si Chen, Wanlong Lei

**Affiliations:** ^1^Department of Anatomy, Zhongshan School of Medicine, Sun Yat-sen University, Guangzhou, China; ^2^Department of Pathology, The Third Affiliated Hospital, Sun Yat-sen University, Guangzhou, China; ^3^Neuroscience Laboratory for Cognitive and Developmental Disorders, Department of Anatomy, Medical College of Jinan University, Guangzhou, China; ^4^Department of Pathology, The Sixth Affiliated Hospital, Sun Yat-sen University, Guangzhou, China; ^5^College of Medicine, Institute of Medical Sciences, Jishou University, Jishou, China; ^6^Department of Human Anatomy, Histology and Embryology, Zunyi Medical University, Zhuhai, China

**Keywords:** cortical glutamatergic terminals, spinal cord, synaptic connection, calretinin interneuron, corticospinal tract

## Abstract

The cerebral cortex innervates motor neurons in the anterior horn of the spinal cord by regulating of interneurons. At present, nerve tracing, immunohistochemistry, and immunoelectron microscopy are used to explore and confirm the characteristics of synaptic connections between the corticospinal tract (CST) and cervical spinal calretinin (Cr) interneurons. Our morphological results revealed that (1) biotinylated dextran amine labeled (BDA+) fibers from the cerebral cortex primarily presented a contralateral spinal distribution, with a denser distribution in the ventral horn (VH) than in the dorsal horn (DH). An electron microscope (EM) showed that BDA+ terminals formed asymmetric synapses with spinal neurons, and their mean labeling rate was not different between the DH and VH. (2) Cr-immunoreactive (Cr+) neurons were unevenly distributed throughout the spinal gray matter, and were denser and larger in the VH than in the DH. At the single labeling electron microscope (EM) level, the labeling rate of Cr+ dendrites was higher in the VH than in the DH, in which Cr+ dendrites mainly received asymmetric synaptic inputs, and between the VH and DH. (3) Immunofluorescence triple labeling showed obvious apposition points among BDA+ terminals, synaptophysin and Cr+ dendrites, with a higher density in the VH than in the DH. (4) Double labeling in EM, BDA+ terminals and Cr+ dendrites presented the same pattern, BDA+ terminals formed asymmetric synapses either with Cr+ dendrites or Cr negative (Cr-) dendrites, and Cr+ dendrites received either BDA+ terminals or BDA- synaptic inputs. The average percentage of BDA+ terminals targeting Cr+ dendrites was higher in the VH than in the DH, but the percentage of BDA+ terminals targeting Cr- dendrites was prominently higher than that targeting Cr+ dendrites. There was no difference in BDA+ terminal size. The percentage rate for Cr+ dendrites receiving BDA+ terminal inputs was lower than that receiving BDA- terminal inputs, and the BDA+ terminal size was larger than the BDA- terminal size received by Cr+ dendrites. The present morphological results suggested that spinal Cr+ interneurons are involved in the regulatory process of the cortico-spinal pathway.

## 1. Introduction

The descending fibers from supraspinal areas form a complex neural network with spinal cord interneurons, which plays an important role in modulating motor outputs and sensory inputs. The corticospinal tract is one of the primary descending fasciculi in the mammalian spinal cord. Its neurons originate from layer V of the sensory motor cortex, which transmits information from the cerebral cortex to the spinal cord (Hicks and D’Amato, 1975; Joosten and van Eden, 1989; Schieber, 2007). In rodents, most fibers in the corticospinal tract cross to the contralateral spinal cord through the pyramid, forming the dorsal funiculus, but a few fibers do not cross, continuing their path on the ipsilateral side and running in the ventral funiculus (Brosamle and Schwab, 1997; Kathe et al., 2014). The corticospinal tract has many different functions. It plays a key role in the descending control of somatosensory afferent input, the regulation of reflex control, and the regulation of interneuron and motor neuron activity in the VH of the spinal cord (Umeda et al., 2010). Therefore, injury to the corticospinal tract caused by spinal cord injury will cause fine movements, such as digital movement and control of distal muscles, to lose considerable flexibility (Lacroix et al., 2004; Kathe et al., 2014). Identifying the types of neurons in the spinal cord that receive inputs from the CST and clarifying the synaptic patterns are critical for understanding the function of the CST. Previous studies have shown that the corticospinal neurons in adult primates can directly contact motor neurons, but some studies have also found that the spinal interneurons of macaques receive input of from the corticospinal descending pathway, exerting pre-synaptic effects on motoneurons to control fine hand movement (Isa et al., 2006; Riddle and Baker, 2010; Witham et al., 2016). Several studies have mapped the distribution of CST axon terminals in different regions of the spinal cord and showed that CST fibers target the dendrites of the distal motoneurons or interneurons in the cat spinal cord (Weidner et al., 2001; Chakrabarty et al., 2009; Chakrabarty and Martin, 2010). In a connection diagram between corticospinal (CS) neurons and various interneurons in the spinal cord, it was found that CST fibers form synaptic connections with proprioceptive and segmental interneurons, which indirectly activate motor neurons in the ventral horn and control voluntary movement in rodents (Ueno et al., 2018).

Cervical spinal calretinin is a member of the calcium-binding proteins (CBPs) family, along with parvalbumin and calbindin, a functional protein family with a special three-dimensional helix circuit helix structure EF hand, which has a high affinity for calcium (Ca^2+^) ions and can consociate specifically with it (Masliukov et al., 2017; Fairless et al., 2019; Gattoni and Bernocchi, 2019). Calcium-binding proteins regulate the level of Ca^2+^ in cells, participate in the release of neurotransmitters and cell motility, maintain the membrane characteristics and electrical activity of neurons, and prevent the death of neurons caused by the release of large amounts of Ca^2+^ in cells (Albuquerque et al., 1999; Wang et al., 2004; Schwaller, 2012). Cervical spinal calretinin neurons are an important component of interneurons in the spinal cord and play an important role in establishing new synaptic contacts between the axons of major ganglion neurons and target organs, pre-ganglionic fibers, and major ganglion neurons (Masliukov et al., 2017). Cervical spinal calretinin neurons can be associated with pain formation by linking harmless tactile information to injurious multisynaptic circuits in the dorsal horn of the spinal cord (Peirs et al., 2015). In addition, Cr neurons can be involved in the reconstruction of spinal motor circuits (Han et al., 2015; Berg et al., 2018). According to the above study, Cr neurons may form a complex local circuit with primary afferent fibers, descending projecting neurons from the superior center or other interneurons and participate in sensory afferents and efferents to regulate the activity of motor neurons (Han et al., 2015; Peirs et al., 2015; Berg et al., 2018; Ueno et al., 2018). Moreover, the development of Cr neurons in the spinal cord of *Celsr3/Fxg1* mice was abnormal because their CST was genetically ablated during early development, indicating that CST is crucial for the function of Cr neurons (Han et al., 2013).

At present, how CST regulates the development and function of Cr neurons in the spinal cord remains unclear. Therefore, we used immunohistochemistry, immunofluorescence and immunoelectron microscopy to explore the location of Cr interneurons, the CST and the characteristics of the synaptic connections between them.

## 2. Materials and methods

### 2.1. Animal experiments and treatment

Twenty-four adult male Sprague-Dawley (SD) rats weighing 250–300 g were used in this study and randomly divided into two groups: a normal group and a primary motor cortex (M1) injection group. There were 12 rats in each group, among which six were used for immunohistochemical staining and six rats for immuno- EM. All rats were obtained from the Center for Experimental Animals of Sun Yat-sen University [Permit no: SYXK (YUE)2015-0107]. The process of animal care, feeding and experimental research was conducted in strict accordance with the National Institutes of Health Guide for the Care and Use of Laboratory Animals and approved by the Animal Care and Use Committee of Sun Yat-sen University (ethical permission no.: Zhongshan Medical Ethics 2014-23). All the experimental animals were kept in the Central Laboratory of Experimental Animals, with room temperature of 22C∼25°C, maintained on a light and dark (12/12 h) cycle, and with good ventilation.

The experimental animals for immunohistochemical staining were anesthetized by intraperitoneal injection of 3% sodium pentobarbital (1 mg/kg), and then perfused with 400 ml of 0.9% saline and followed by 400 ml of 4% paraformaldehyde-15% saturated picric acid (in 0.1 M phosphate buffer, pH 7.4). Rats for immuno-EM were perfused by the abovementioned method, but 0.6% glutaraldehyde was added to the fixative. The cervical spinal cord of the rats (cervical 5 ∼ cervical 7) was removed and immersed in fixative (4% paraformaldehyde-15% saturated picric acid) overnight at 4°C. The cervical spinal cord tissue was sectioned continuously at 50 μm by the automatic vibratome (catalog no. VT1200S, Leica, Weztlar, Germany).

### 2.2. Neural tracing

After intraperitoneal anesthesia with 3% sodium pentobarbital (1 mg/kg), the rats were fixed on the operating table in the prone position, and then placed on a stereotaxic apparatus (catalog no. 60191, Stoelting Co., IL, USA). The skull was exposed and a hole was drilled to expose the right M1 area. A Hamilton microsyringe was used to inject a total of 3 μL of BDA solution (10,000 MW, 10% dissolved in 0.1 M PBS, catalog no. D1956 Molecular Probes, MA, USA) into the M1 area of rats, which was identified with the rat brain atlas [(M1 coordinates): anterior-posterior (AP): 3.7 mm; medial-lateral (ML): −2.2 mm; and dorsal ventral (DV): −1.5 mm]. The tracer was injected three times with 5 min intervals to minimize tracer contraflow along the injection tract and promote cortex absorption of the tracer. Before the micropipette withdrawal, it stayed in place for 15 min.

### 2.3. Single-labeling experiment for light microscopy (LM) and EM

#### 2.3.1. LM experiment

The spinal cord sections injected with BDA 10 kDa were placed in 0.3% H_2_O_2_ at room temperature for 30 min. Liquid A and liquid B in the ABC kit (1:100, catalog no. PK-6100, Vector Labs, San Francisco, CA, USA) were mixed in equal volumes and incubated at 4°C for 24 h. The sections from the normal group were pre-treated with 0.3% H_2_O_2_, incubated at room temperature for 30 min, and then incubated at 4°C for 48 h without (as negative control samples) or with the primary antibody: rabbit anti-Cr antibody (1:800, catalog no. AB5054, Millipore, MA, USA) diluted with 0.1 M PB containing 1% bovine serum albumin (BSA) and 0.3% Triton X-100. The sections were rinsed and incubated with the secondary antibody: biotinylated anti-rabbit IgG (1:100, catalog no. BA2001, Vector Labs, San Francisco, CA, USA) at room temperature for 2 h, followed by incubation in ABC (1: 200, catalog no. PK-6100, Vector Labs, San Francisco, CA, USA) at room temperature for 2 h. Sections from the normal group and M1 injection group were treated with 3, 3’-diaminobenzidine (DAB, 0.05% in 0.1 M PB, catalog no. D5637, Sigma, MO, USA) for coloration. Between each step above, sections were rinsed with 0.1 M PB 3 times for 5 min each time. After coloration, the sections for immunohistochemistry were mounted onto gelatin-coated slides, dried naturally, dehydrated by conventional gradient alcohol, cleared with xylene and covered with neutral balsam. After air drying, images were collected under a light microscope (LM).

#### 2.3.2. EM experiment

The sections for EM were rinsed in sodium cacodylate buffer (0.1 M, catalog no. 6131-99-3, Xiya reagent, Chengdu, China) after visualization with BDA, and post-fixed in 2% OsO4 (osmium tetroxide, OsO4; catalog no. 18456, PELCO, CA, USA) for 1 h, dehydrated in a graded series of ethyl alcohols, and impregnated with 1% uranyl acetate in 100% alcohol and flat-embedded in Epon 812 resin (catalog no. 18010, PELCO, CA, USA). Ultrathin sections were cut with an ultramicrotome (catalog no. EM UC6, Leica Microsystems, Weztlar, Germany). These sections were stained with 0.4% lead citrate and 4.0% uranyl acetate again, and finally viewed with an EM (Tecnai G2 Spirit Twin, FEI; Thermo Fisher Scientific, Inc., MA, USA).

### 2.4. Triple-labeling and double-labeling experiments for LM and EM

#### 2.4.1. Double-labeling experiment for LM

For analysis of the density of BDA+ fibers and Cr+ neurons in the cervical spinal cord, spinal cord sections injected with BDA and normal sections were double-labeled for BDA and NeuN (neurons). The BDA+ fibers and Cr+ neurons were immunostained as described, but with a nickel-intensified DAB reaction (DAB solution containing 0.04% nickel ammonium sulfate), and then incubated at 4°C for 48 h without (as negative control samples) or with the following primary antibody: mouse anti-NeuN antibody (1:500, catalog no. MAB377, Millipore, MA, USA). The sections were rinsed and incubated with the secondary antibody: biotinylated anti-mouse IgG (1:100, catalog no. BA2001, Vector Labs, San Francisco, CA, USA) at room temperature for 2 h. The sections were stained with DAB solution. For each step above, 0.1 M PB was rinsed three times, for 5 min each time.

#### 2.4.2. Triple-labeling experiment for LM

Spinal sections for BDA injection were incubated with the primary antibodies: rabbit anti-Cr antibody, mouse anti-synaptophysin antibody (1:1000, catalog no. SAB4200544, Sigma, MO, USA) and then incubated without (as negative control samples) or with a mixture of anti-rabbit IgG (AF647, 1:400, catalog no. A-21244 Invitrogen, MA, USA), anti-mouse IgG (AF488, 1:400, catalog no. A11029, Thermo Fisher Scientific, MA, USA) and Cy3-streptacidin antibody (1:400, catalog no. 212-166-168, Jackson ImmunoResearch, PA, USA) at room temperature for 1 h. Rinsed with 0.1 M PB for three times between each step for 5 min each time, sealed with anti-fluorescence quenching agent (catalog no. P36930, Invitrogen, MA, USA), and observed and imaged under a laser scanning confocal microscope (LSCM, catalog no. Eclipse Ni-E, Nikon, Tokyo, Japan).

#### 2.4.3. Double-labeling experiment for EM

For detection of the synaptic connections between cortical glutaminergic terminals and spinal Cr+ neurons, spinal sections injected with BDA10k were immunolabeled as described above but with a nickel-intensified DAB reaction (DAB solution containing 0.04% nickel ammonium sulfate), incubated at 4°C for 48 h in a rabbit anti-Cr primary antibody, rinsed and incubated with secondary antibodies (biotinylated anti-rabbit IgG) at room temperature for 2 h. The sections were stained with DAB solution. For each step above, three rinses with 0.1 M PB for 5 min were performed.

### 2.5. Data collection and statistical analysis

We divided the gray matter of the spinal cord into the dorsal horn and ventral horn by referring to previous articles (Gauriau and Bernard, 2004; Islam et al., 2017) and mainly focused on layers V, and VI of the dorsal horn and layers VII, and VIII of the ventral horn. The electron microscope samples were of course taken from this area.

For the LM data, five line segments (100 μm) were randomly drawn in each zone, and the intersection points of immunostained fibers with this line were counted as the density of BDA+ fibers. The density of Cr+ neurons was counted in five non-overlapping squares (100 × 100 μm) within the gray matter, and taken the mean of the major axis and minor axis was taken as the soma size of Cr+ neurons. For the triple-labeling experimental result, the BDA+ fibers (red) formed clear apposition points (white) with Syn1 (green) and Cr+ neurons (blue) in the gray matter, and the density of apposition points was the number of apposition points in five random squares of 100 μm^2^ within the gray matter in each image. Each mouse was photographed three times, and the mean value of these sections was used as the statistical data for each rat.

For the EM data, the analysis was based on 30 EM images per animal. The widest diameter parallel to and 0.1 μm before the pre-synaptic membrane of the terminals was measured as their size. Many immunoreactivity products were visible in BDA+ terminals and Cr+ neurons, and the dendrites were identified by their size, oval or elongated shape, and the presence of microtubules and mitochondria. The thicknesses of the pre-synaptic and post-synaptic membrane of symmetrical synapses were essentially equal, but the post-synaptic membranes of asymmetric synaptic contacts was significantly thicker than the pre-synaptic membrane, as shown by the thick post-synaptic density (PSD). For detailed count methods please refer to our previous paper (Lei et al., 2013; Ma et al., 2014a,b; Zheng et al., 2018, 2021). The investigators were blinded for the morphological studies.

All data are expressed as the mean ± standard deviation (SD). All data were analyzed using SPSS 20.0 software to test normality. Only the data of apposition density did not conform to a normal distribution, and we conducted a Wilcoxon rank sum test for these data separately. The *t*-test was used to analyze the data (the density of BDA+ fibers, the BDA+ terminal size, the labeling rate of BDA+ terminals, the density of Cr+ neurons, the Cr+ neurons size, the labeling rate of Cr+ dendrites and the size of asymmetric terminals targeting Cr+ dendrites) between DH and VH. The data of the ratio of synaptic connections formed by BDA+ terminals and the dendrites of interneurons, the size of synaptic connections formed by BDA+ terminals and the dendrites of interneurons, the ratio of synaptic connections formed by the dendrites of Cr+ neurons and axon terminals, the size of synaptic connections formed by axon terminals and Cr+ neurons were analyzed by one-way ANOVA. All graphs were generated using GraphPad Prism 8 (GraphPad software, San Diego, CA, USA). *P* < 0.05 was considered statistically significant.

## 3. Results

### 3.1. Distribution and ultrastructural characteristics of cortical glutamatergic terminals in the cervical spinal gray matter

**FIGURE 1:**
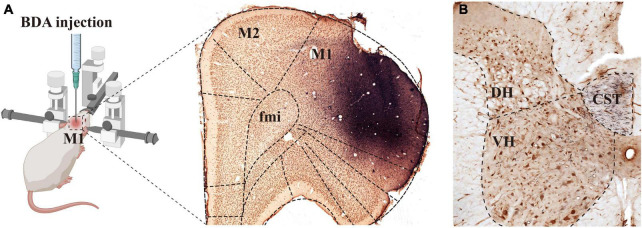
Biotinylated dextran amine (BDA) was injected into M1 and the labeled fibers were distributed in the spine. Image [**(A)**, left panel] shows the rats that were fixed on the operating table in the prone position and placed on a stereotaxic apparatus, and BDA solution was injected into the M1 region of the rat cerebral cortex. The (right panel) in image **(A)** shows the immuno-light microscopy (LM) images at the injection sites, BDA solution (blue) was absorbed by pyramidal cells (brown) of the M1 region. **(B)** Shows that BDA+ fibers crossed to the ventral region of the dorsal funiculus on the opposite side of the injection site, the crossed CST extended into the spinal gray matter, and most of them were distributed in the deep dorsal horn and the ventral horn. M1, primary motor cortex; M2, second motor cortex; fmi, forceps minor of the corpus callosum; CST, corticospinal tract; DH, dorsal horn; VH, ventral horn.

**FIGURE 2:**
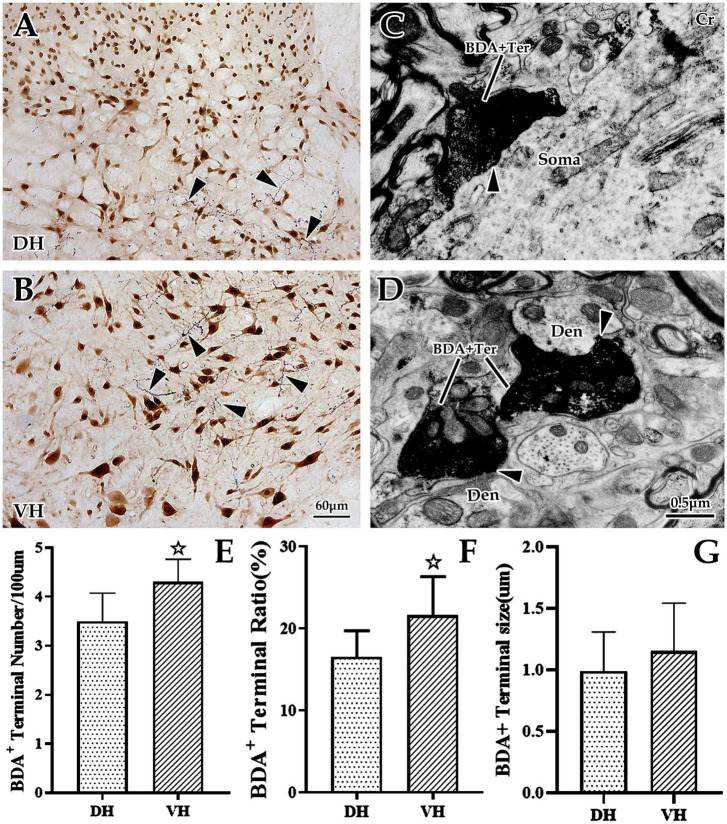
The distribution and ultrastructural characteristics of cortical glutamatergic terminals in the cervical spinal gray matter. **(A,B)** Show cortical glutamatergic terminals labeled by biotinylated dextran amine (BDA) injection (blue) and spinal neurons immunolabeled by NeuN (brown). BDA+ fibers are observed at the dorsal horn (DH) **(A)** and ventral horn (VH) **(B)**, and these beaded BDA+ fibers had abundant branches (△). Electron microscope (EM) showed that BDA+ terminals were oval or circular, with a large number of medium-sized vesicles. These positive terminals made asymmetric (excitatory) synaptic connections either with somas **(C)** or with dendrites **(D)**. In the above panels, the DH and VH were short of the dorsal horn and the ventral horn. Den, dendrite; Ter, terminal; Soma, cell body. **(A,B)** Show the same magnification with a scale bar = 60 μm. **(C,D)** Show the same magnification, and the scale bar is shown in the right lower corner of **(D)**. Histogram **(E)** shows the density of BDA+ fibers in the DH and VH for immuno-light microscopy (LM) experiments. Histograms **(F,G)** show the labeling rate and size of BDA+ terminals in the DH and VH for immuno-EM experiments, respectively. In the above panels, the DH and VH were short of the dorsal horn and the ventral horn. Den, dendrite; Ter, terminal; Soma, cell body. ^☆^*P* < 0.05, compared between the DH and VH.

Our previous studies confirmed that BDA10k, an anterograde tracer, perfectly labeled neurons and their projections, especially axon terminals. In the present experiment, we injected BDA10k into the cortical M1 area to label cortical glutamatergic terminals projecting to cervical spinal gray matter (Figures 1A, B). LM observation revealed that a large number of BDA+ fibers accumulated in the contralateral dorsomedial funiculus near the basilar region of the dorsal horn (Figures 2A, B), extending into the DH and VH. BDA+ fibers exhibited a beaded appearance with uneven thickness and irregular branches (Figures 2A, B). A few BDA+ fibers were observed in the spinal gray matter ipsilateral to the cortical injection site. The results showed that the average density of BDA+ fibers in the contralateral spinal gray matter was 3.90 ± 0.53/100 μm, and further comparative results demonstrated a significant difference in BDA+ fiber density (312 fibers in 6 rats,140 fibers in the DH, 172 fibers in the VH, *P* < 0.05, Figure 2E) between the DH (3.50 ± 0.58/100 μm) and VH (4.30 ± 0.47/100 μm). The BDA+ fibers of each mice in the dorsal horn were 4.40, 3.80, 5.20, 4.60, 4.20, 4.00/100 μm, and their corresponding BDA+ fibers density in the ventral horn were 3.40, 3.20, 4.20, 3.80, 3.60, 2.80/100 μm.

Next, EM was applied to explore the ultrastructural characteristics of BDA+ terminals, and the results revealed that BDA+ terminals were mostly oval or round in appearance, containing a large number of evenly distributed, medium-sized, clear, spherical vesicles. These BDA+ terminals made asymmetric synaptic contact with the dendrites and somas of spinal neurons (Figures 2C, D). Further data showed that the average labeling rate of BDA+ terminals (BDA+ terminals/total terminals, 529 terminals in six rats, 282 BDA+ terminals in the DH, 247 BDA+ terminals in the VH) was 19.08 ± 1.32%, which was 16.53 ± 1.07% in the DH and 21.63 ± 1.57% in the VH, and this difference was significant (*P* < 0.05, Figure 2F). The measurement results showed that the mean size of these BDA+ terminals (105 BDA+ terminals in 6 rats, 49 BDA+ terminals in the DH, 56 BDA+ terminals in the VH) was 1.07 ± 0.36 μm, and the BDA+ terminal size between the DH (0.99 ± 0.32 μm) and VH (1.15 ± 0.39 μm) was not significantly different (*P* > 0.05, Figure 2G).

### 3.2. Morphological structure and distribution of cervical spinal Cr+ neurons

**FIGURE 3:**
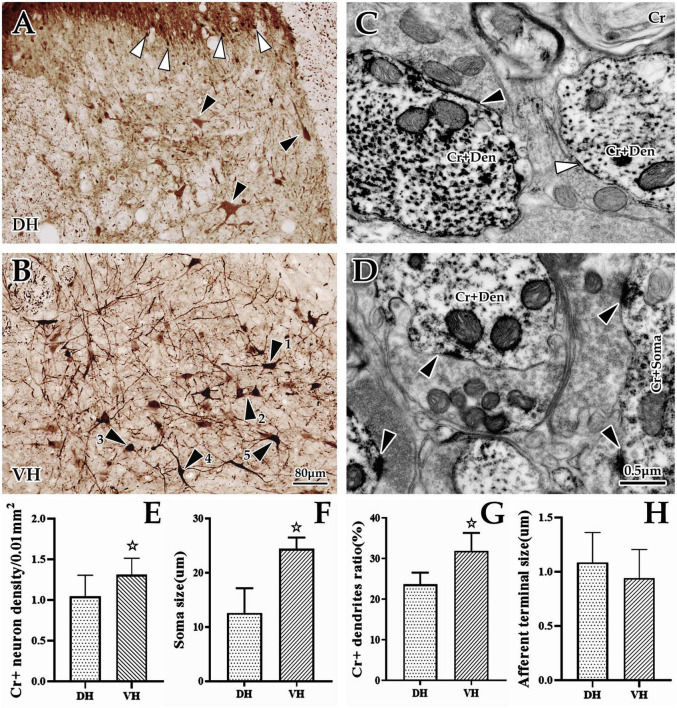
Morphological and distribution characteristics of spinal Cr+ neurons. The panels above show immunohistochemical single labels of Cr+ neurons (brown) in the dorsal horn (DH) **(A)** and ventral horn (VH) **(B)**, in which Cr+ neurons showed a densely banded distribution in the superficial dorsal horn (layers I and II), with tiny spherical somas and rare processes (▲), but in the deep dorsal horn (layers III–VI), they were sparsely distributed, with larger irregular cell bodies and more processes [▲, **(A)]**. In the VH **(B)**, Cr+ neurons showed a dense distribution with larger cell bodies and bushy processes. These Cr+ neurons appeared to have various shapes: some of them were polygonal and pyramidal (▲1 and ▲2), and others were spherical, fusiform and semilunar (▲3, ▲4, and ▲5). **(C,D)** Show the ultrastructure of Cr+ neurons at the immuno-electron microscope (EM) level. A large number of Cr+ dendrites **(C)** and somas **(D)** with asymmetric synaptic inputs (▲) were clearly visible, but some were visible for symmetric synaptic inputs [**(C)**, ▲]. The abbreviations in this figure are the same as in Figure 2. Light microscope (LM) images were obtained at the same magnification; scale bar = 80 μm. The magnification of the EM images is the same as the scale bar in the right lower corner of **(D)**. Histograms **(E,F)** show the density and soma size of Cr+ neurons in the DH and VH for immuno-LM experiments, respectively. Histograms **(G,H)** show the labeling rate and size of Cr+ dendrites in the DH and VH for immuno-EM experiments, respectively. ^☆^*P* < 0.05, compared between the DH and VH.

Immunohistochemical labeling revealed that Cr+ neurons presented a densely banded distribution in the superficial DH (layers I and II), with small, round somas and few projections. However, Cr+ neurons were sparsely distributed in the deep DH (layer III-VI), whose somas were markedly larger than those of the superficial layer, with an irregular appearance and more processes (Figures 3A, B). In the VH, Cr+ neurons were densely distributed, with obviously large somas and abundant projections, in which these somas presented a pleomorphic appearance: some were polygonal and pyramidal, while others were spherical, fusiform and semilunar (Figures 3A, B). Enumeration data showed that the average density of spinal Cr+ neurons was 1.18 ± 0.23/0.01 mm^2^, 1.04 ± 0.26/0.01 mm^2^ in the DH, and 1.31 ± 0.20/0.01 mm^2^ in the VH, with a significant difference between them (*P* < 0.05, Figure 3E). Measurement and statistical results revealed that the average size of these Cr+ neurons was 16.88 ± 1.48 μm and 12.57 ± 1.28 μm in the DH and 24.40 ± 0.74 μm in the VH, which was distinctly larger in the VH than in the DH (*P* < 0.05, Figure 3F).

Further investigation using immuno-EM showed a large number of Cr+ particles dispersed in the soma and processes (Figures 3C, D). These Cr+ dendrites appeared to have different thicknesses, but it was clear that most of them received asymmetric (excitability) synaptic inputs with a few symmetric (inhibitory) synapses (Figures 3C, D). The average labeling rate (Cr+ dendrites/total dendrites, 733 dendrites in six rats, 449 dendrites in DH, 284 dendrites in VH) of Cr+ dendrites was 27.79 ± 1.22%, which was 23.66 ± 0.96% in the DH and 31.92 ± 1.47% in the VH, and there was a significant difference between them (*P* < 0.05, Figure 3G). An average of 85.97 ± 3.68% of Cr+ dendrites received asymmetric synaptic inputs (data were obtained from six animals: 196 Cr+ dendrites received synaptic inputs, 99 Cr+ dendrites received synaptic inputs in DH, 97 Cr+ dendrites received synaptic inputs in VH), which was 83.57 ± 2.04% in the DH and 88.36 ± 5.32% in the VH, but there was no difference between them (*P* > 0.05). Measurement results showed that the mean size of asymmetric terminals targeting Cr+ dendrites (164 Cr+ dendrites in six rats, 80 Cr+ dendrites in the DH, 84 Cr+ dendrites in the VH) was 1.03 ± 0.09 μm, and 0.96 ± 0.40 μm for the DH, and 1.09 ± 0.35 μm for the VH, with no significant difference observed (*P* > 0.05, Figure 3H).

### 3.3. Morphological examination of the synaptic connection of cortical glutamatergic terminals with spinal Cr+ dendrites using LM and EM

#### 3.3.1. Immunofluorescence triple labeling experiment

**FIGURE 4:**
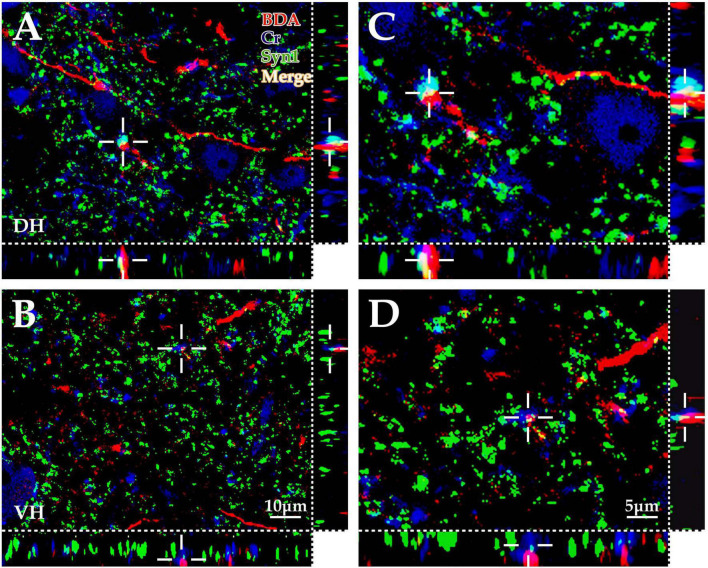
Immunofluorescence exploration of the synaptic connections between BDA+ fibers and spinal Cr+ neurons. The panels above show that the cortex-spinal cord projected fibers labeled by biotinylated dextran amine (BDA) (red) formed clear appositions (white) with synaptophysin (Syn1) (green) and Cr+ neurons (blue) in the gray matter by immunofluorescence triple labeling. As shown in the above panels, a large number of BDA+ fibers (red) were observed in the dorsal horn (DH) **(A,C)** and ventral horn (VH) **(B,D)**, which was similar to the morphological appearance developed by DAB shown in Figure 1. Moreover, a large number of Cr+ dendrites and somas (blue) were observed, and Syn1-positive structures presented granulate and patch appearances (green). The apposition points (white) of three structures were observed with three-dimensional recomposition, which was labeled with the cross symbol (+). The abbreviations in this figure are the same as in Figure 2. **(A,B)** Show the same magnification with a scale bar = 10 μm. **(C,D)** Are the high magnification views of **(A,B)**, respectively. The cross symbol (+) in **(C,D)** is the same apposition in **(A,B)**, respectively. The magnifications of **(C,D)** are the same, scale bar = 5 μm.

**FIGURE 5:**
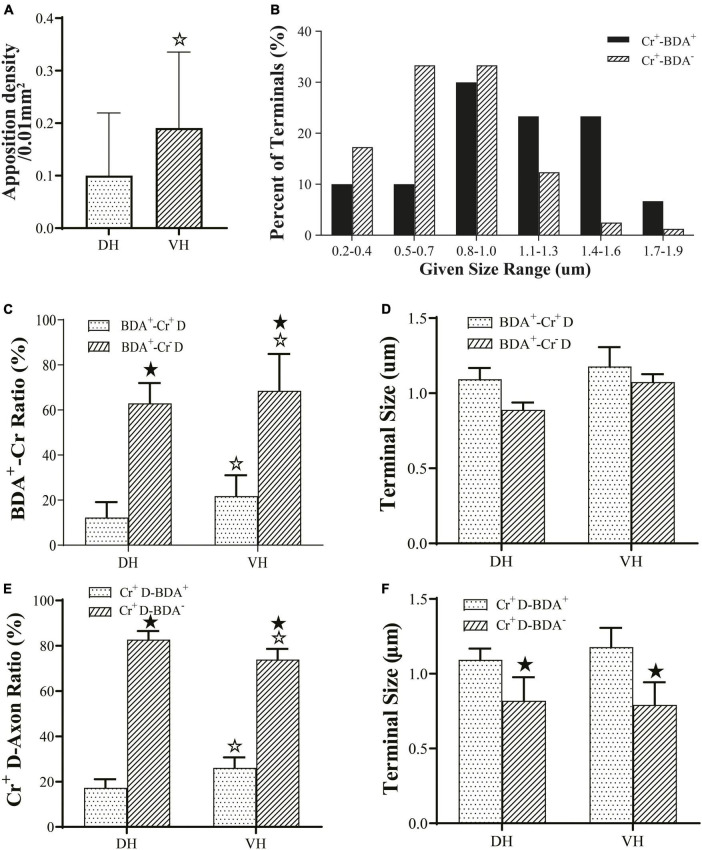
Connection characteristics between fibers from the M1 region and CR+ neurons of the cervical spine. Histogram **(A)** shows the density of apposition points between biotinylated dextran amine (BDA+) fibers, Cr+ neurons and synaptophysin-positive (syn1+) structures. Histogram **(B)** shows the size-frequency distributions of BDA+ and BDA− terminals received by Cr+ dendrites for immuno-electron microscope (EM) experiments. The BDA+ terminals received by Cr+ dendrites mainly ranged in size from 0.8 to 1.6 μm, and the BDA- terminals received by Cr+ dendrites ranged in size from 0.2 to 1.0 μm range. Histogram **(C)** shows the ratio of synaptic connections formed by BDA+ terminals and the dendrites of interneurons. Histogram **(D)** showed the size of synaptic connections formed by BDA+ terminals and the dendrites of interneurons. Histogram **(E)** shows the ratio of synaptic connections formed by the dendrites of Cr+ neurons and axon terminals. Histogram **(F)** shows the size of synaptic connections formed by axon terminals and Cr+ interneurons. In the above panels, the DH and VH were short of the dorsal horn and the ventral horn. Cr^+/–^ D, dendrite of Cr+ or Cr- neurons; ^★^*P* < 0.05, compared between the Cr+-BDA+ group and the Cr+-BDA-group; ^☆^*P* < 0.05, compared between DH and VH.

The present morphological experiment aimed to examine the positional relationship between cortical glutamatergic terminals and cervical spinal Cr+ neurons at the LM level. Laser confocal microscopy exploration revealed that a large number of cortical glutamatergic axons labeled by BDA10k injection exhibited a fibrous appearance in the cervical spinal gray matter. Cr− immunolabeled structures were primarily a large number of dense dendrites and somas, while synaptophysin-positive (syn1+) structures presented a granular or patchy appearance (Figures 4A, B), showing clear appositions with 3D reconstruction (Figures 4C, D). Statistical data demonstrated that the average density for apposition points was 0.15 ± 0.03/0.01 mm^2^ in the contralateral spinal gray matter, with 0.10 ± 0.02/0.01 mm^2^ in the DH and 0.19 ± 0.03/0.01 mm^2^ in the VH, and this difference was significant (*P <* 0.05, Figure 5A).

#### 3.3.2. Double labeling exploration at the EM level

**FIGURE 6:**
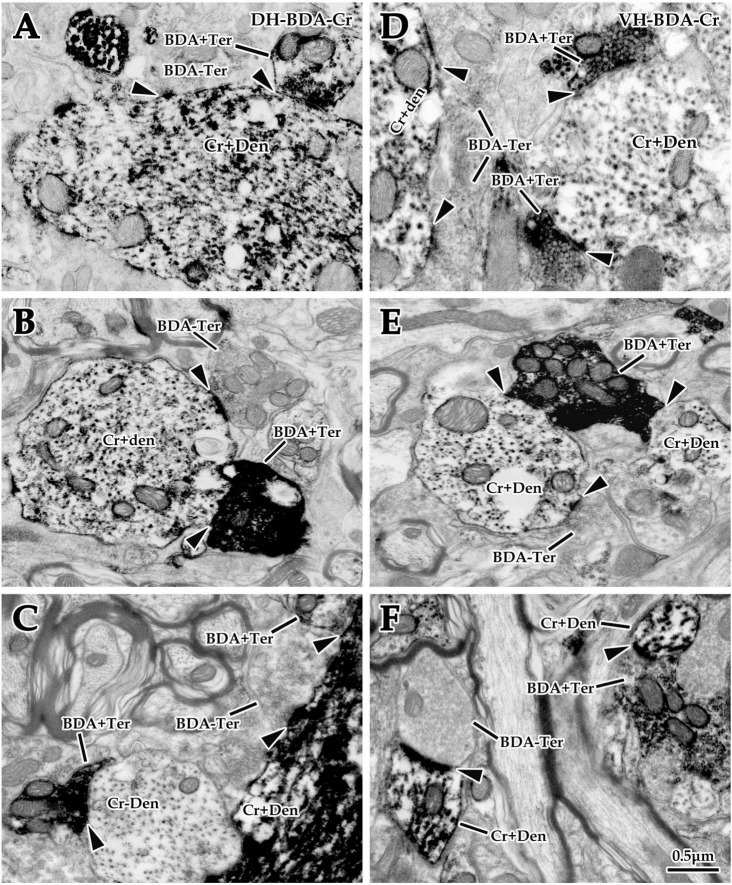
Double labeling of synaptic connections between cortical glutaminergic terminals and spinal calretinin (Cr) neurons for immuno-electron microscope (EM) experiments. Immuno-EM double-labeling above shows that a large number of biotinylated dextran amine (BDA+) terminals were observed in the dorsal horn (DH) **(A–C)** and ventral horn (VH) **(D–F)** in the spinal gray matter, which made typical asymmetric synaptic connections (▲) with either Cr+ **(A–F)** or Cr- **(C)** dendrites. Moreover, a mass of Cr+ dendrites and somas were observed in the same region, which received excitatory synaptic inputs of either BDA+ or BDA- terminals (▲). The abbreviations in this figure are the same as in Figure 2. All panels are the same magnification with scale bar = 0.5 μm.

Double labeling of immuno-EM is an ideal and intuitive technique to confirm synaptic connections. In the present experiment, BDA10k was injected into the cortical M1 area to label cortical glutamatergic terminals extending into the spinal gray matter, and immunohistochemistry was used to label spinal Cr+ neurons. Immuno-EM exploration revealed that BDA+ terminals were mostly oval or spherical in appearance and contained a large number of synaptic vesicles. BDA+ terminals were distributed in both the DH and VH (Figures 6A–F), and Cr+ dendrites and somas were clearly observed in the same figure (Figures 6A–F). These BDA+ terminals made asymmetric synaptic connections with either Cr+ dendrites or Cr- dendrites in both the DH and VH. Cr+ dendrites and somas received either BDA+ or BDA unlabeled (BDA-) terminal synaptic inputs (Figures 5B, 6A–F), but the present experiment primarily focused on the synaptic connection between BDA+ terminals and Cr+ dendrites.

#### 3.3.3. Morphological characteristics of BDA+ terminal targeting in EM double labeling experiments

Comparative data (from six animals, 105 BDA+ terminals, 49 BDA+ terminals in the DH, 56 BDA+ terminals in the VH) on BDA+ terminal targeting showed that the average 16.51 ± 5.75% of BDA+ terminals targeted onto Cr+ dendrites, with 12.85 ± 6.20% in the DH and 20.17 ± 5.29% in the VH, and there was a significant difference between them (*P <* 0.05, Figure 5C). Measurement results revealed that BDA+ terminals targeting Cr+ dendrites exhibited a mean size of 1.13 ± 0.27 μm and 1.08 ± 0.27 μm in the DH and 1.18 ± 0.30 μm in the VH, with no difference between them (*P* > 0.05, Figure 5D). Enumeration data demonstrated that 65.38 ± 5.03% of BDA+ terminals targeted Cr- dendrites, which was 62.84 ± 4.84% in the DH and 67.92 ± 5.21% in the VH with a significant difference between them (*P* < 0.05, Figure 5C). Additionally, the sizes of the BDA+ terminal targeting Cr- dendrites were 0.98 ± 0.31 μm, 0.88 ± 0.35 μm for the DH and 1.07 ± 0.24 μm for the VH, with no significant difference observed between them (*P* > 0.05, Figure 5D). Further comparison demonstrated that the percentage of BDA+ terminals targeting Cr- dendrites (65.38 ± 5.03%) was higher than that targeting Cr+ dendrites (16.51 ± 5.75%, *P* < 0.05, Figure 5C), but no difference was observed in BDA+ terminal size between Cr- dendrites (0.98 ± 0.31 μm) and Cr+ dendrites (1.13 ± 0.27 μm, *P* > 0.05, Figure 5D).

#### 3.3.4. Morphological characteristics of Cr+ dendrites receiving synaptic inputs in EM double labeling experiments

Double labeling data of immuno-EM showed that a mean of 21.67 ± 4.20% Cr+ dendrites received synaptic inputs of BDA+ terminals, which was 17.29 ± 3.78% in the DH and 26.05 ± 4.61% in the VH, with a significant difference between them (*P* < 0.05, Figure 5E), and these BDA+ terminal sizes are shown above. Further exploration revealed that 78.33 ± 1.90% of Cr+ dendrites received BDA- synaptic inputs, which was 82.71 ± 1.89% in the DH and 73.95 ± 1.90% in the VH, and there was a significant difference between them (*P* < 0.05, Figure 5E). The BDA- terminal mean size was 0.81 ± 0.19 μm, which was 0.82 ± 0.21 μm in the DH and 0.79 ± 0.19 μm in the VH, and there was no difference between them (*P*> 0.05, Figure 5F). Comparative results showed that the percentage of Cr+ dendrites receiving BDA+ terminal inputs (21.67 ± 4.20%) was markedly less than that receiving BDA- terminal inputs (78.33 ± 1.90%, *P* < 0.05, Figure 5E), in which the size of the BDA+ terminal (1.13 ± 0.27 μm) received by Cr+ dendrites was significantly larger than that of the BDA- terminal received by Cr+ dendrites (0.81 ± 0.19 μm, *P* < 0.05, Figure 5F). The frequency analysis demonstrated that 76.67% of the BDA+ terminals received by Cr+ dendrites had a size from 0.8 to 1.6 μm, and 83.94% of the BDA- terminals received by Cr+ dendrites had a size of 0.2–1.0 μm (Figure 5B).

## 4. Discussion

### 4.1. Morphological and functional characteristics of corticospinal projections

In mammals, the axons of the CST originate from pyramidal cells in the inferior region of cortical layer V in the M1, primary somatosensory cortex (S1) and premotor area, ultimately terminating in different regions of the spinal cord gray matter, which is a complex descending pathway that participates in the regulation of sensory information, spinal reflexes and autonomic movement control, especially of fine motion (Alstermark and Pettersson, 2014; Kondo et al., 2015; Zhang et al., 2019). BDA is a dextran amine (DA) labeled by biotin. This molecule can be used as an anterograde and retrograde chemical tracer to study the structure of neurons and axon terminals, and it is the gold standard for many experiments for localizing neural anatomy pathways (Veenman et al., 1992; Reiner et al., 2000). In this study, we used a microinjection method to inject BDA at multiple points in the M1 region of the rat cerebral cortex. BDA was absorbed by pyramidal cells and transported to the level of the cervical spinal cord along the axon of the corticospinal tract. At the LM level, the results showed that BDA+ fibers crossed to the ventral region of the dorsal funiculus on the opposite side of the injection site, while only a few fibers terminated in the ipsilateral gray matter of the spinal cord. Then, the crossed CST extended into the spinal gray matter, and most of them were distributed in the deep dorsal horn and layers VII–VIII of the ventral horn. The projection pattern of CST observed here is distinct from that of some previous studies, which showed that the distribution of CST fibers in the dorsal horn of the spinal cord was denser and sparse on the ventral side (Casale et al., 1988; Valtschanoff et al., 1993; Du Beau et al., 2012). Our current observations are more similar to those of Bareyre et al. (2002) and Suter and Shepherd (2015). Similarly, BDA+ fibers were not distributed in all laminae of the spinal cord gray matter or in the superficial layer of the dorsal horn, and more fibers terminated in the ventral horn than in the dorsal horn. This finding is because the cortical region of the origin of CST axons determines the termination region of CST axons in the spinal gray matter, and the descending CS projections in different cortical regions may have different functions in autonomous motor control. The projections from the primary and secondary sensory cortex are more distributed in the dorsal horn of the spinal gray matter, which may be involved in the control of sensory afferents. Projections from the motor cortex are more widely distributed in the ventral horn, which tends to dominate the motor control of the limbs. These fibers integrate motor output and sensory input in the spinal cord in a coordinated way by connecting with different spinal neurons (Olivares-Moreno et al., 2017). To further observe the morphological structure of BDA+ terminals, we used EM technology to explore the ultrastructure of corticospinal neurons. At the EM level, the distribution density of BDA+ terminals was consistent with that of LM. There are many medium-sized spherical transparent vesicles at the end of BDA+ terminals, which form asymmetric synaptic connections with dendrites. The distribution range of the axon size is similar to that reported in previous studies (Dunkerley and Duncan, 1969; Yang and Lemon, 2003). Moreover, the experimental results revealed that terminals located in the ventral horn are slightly larger than those in the dorsal horn, presumably because the CS axons derived from the M1 area perform separate and different functions related to sensation and movement in different regions of the gray matter, and the sizes of axons in different regions are different (Smith and Alloway, 2013).

### 4.2. Morphological and functional characteristics of spinal Cr interneurons

Cr-immunoreactive neurons are one subset of CBP-positive neurons in the spinal cord (Berg et al., 2018). The morphology and distribution of Cr+ neurons exhibited regional differences, and Cr+ neurons in the cervical and lumbar cords were markedly larger and more abundant than those in the thoracic cord (Chen et al., 2016). In this study, the morphological characteristics and distribution pattern of Cr+ neurons in the cervical spinal gray matter were explored. The results revealed that Cr+ neurons are distributed in each layer of the cervical gray matter in rats and are primarily located in layers II and VI-VIII. There were significant differences in the morphology of the positive neurons in the dorsal and ventral horns, and the morphology of Cr+ neurons in the spinal horn was morphologically distinct from those we previously explored in the striatum. The Cr+ neurons in the striatum were small in size, oval-shaped or triangular in appearance, possessed very few dendrites, and were sparsely distributed (Ma et al., 2014a). In addition, Cr+ neurons in the ventral horn were significantly larger than those in the dorsal horn at the LM level, consistent with a previous study (Anelli and Heckman, 2005). However, there are few reports regarding the ultrastructure of Cr+ neurons, so we used EM technology to further explore the morphological characteristics of Cr+ neurons. The EM results revealed that the positive electron-dense particles in Cr+ neurons were free in the cytoplasm of their dendrites and somas, and most of them were observed in dendrites. Most dendrites of Cr+ neurons receive axons to form asymmetric synaptic connections, and the number of dendrites in the ventral horn was markedly higher than that in the dorsal horn, consistent with our LM observation that the Cr+ neurons in the dorsal horn do not have processes or have fewer and shorter processes, while the ventral horn has more and longer processes. The superficial layer of the spinal dorsal horn is the key region regulating and integrating somatosensory information, such as nociception, pruritus, light touch and thermal perception (Bourane et al., 2015; Todd, 2017). However, we observed a dense distribution of Cr+ neurons in layer II, indicating that Cr+ neurons participate in the transmission of spinal sensory signals. They can form a complex connection network with other interneurons in the spinal dorsal horn and act as amplifiers of incoming sensory inputs, which receive nociceptive inputs, amplify the pain signal, and generate mechanical allodynia and nocifensive responses (Smith et al., 2016, 2019; Gutierrez-Mecinas et al., 2019). In addition, a large number of Cr+ neurons were found in the contralateral layer VIII and the ipsilateral deep layer (layers V, VI) of the dorsal horn after injecting the b-subunit of cholera toxin (CTb), a retrograde tracer, into the motor nucleus of the spinal cord in rats, and the researchers believed that that some Cr+ neurons in layers VII and VIII were propriospinal neurons (PSNs) (Liu et al., 2010; Brockett et al., 2013). Hence, Cr+ neurons might be related to limb proprioception, participate in the conduction of information from the muscle spindle, tendon and tactile receptor, transmit nerve impulses to spinal motor neurons to coordinate limb movement and adjust posture by exciting motor neurons (Alvarez et al., 2005; Ichikawa et al., 2005; Morona et al., 2006; Zhang et al., 2014, 2016). The results of this study reveal that the number of Cr+ neurons in the ventral horn is greater than that in the dorsal horn, indicating that Cr+ neurons may have more connections with motor control.

### 4.3. Synaptic features of cortical glutamatergic terminals with cervical spinal calretinin neurons

In this study, the synaptic connection between corticospinal projection-positive terminals and Cr+ neurons in the spinal cord was studied using immunofluorescence triple labeling and immunoelectron microscopy double labeling. There have been few previous reports on this topic, especially in the cervical spinal cord. Synaptophysin, a pre-synaptic structural component, is widely localized in the synaptic vesicles of membrane proteins, and many experiments have detected the extent of synaptic connections by labeling it (Piorkowska et al., 2014; Kamiyama et al., 2015; Femi-Akinlosotu et al., 2019). This finding is different from the Chakrabarty observation of connections between intermediate neurons and corticospinal projections only on a plane. In this study, we used synaptophysin to demonstrate that the CST projects to Cr+ interneurons to form synaptic connections (Chakrabarty and Martin, 2010). The number of Cr+ neurons in the ventral horn receiving corticospinal projections was greater than that in the dorsal horn. These results provide basic morphological data for the synaptic connections of CST fibers in the spinal cord gray matter and further double labeled the corticospinal projection and Cr+ neurons at the ultrastructural level. We found that the number of positive synaptic connections in the ventral and dorsal horn at the EM level was consistent with that in light microscopy, which indicated that there was a direct synaptic connection between the CST and the Cr+ neurons in the cervical gray matter of the spinal cord, and the CST originating from the motor cortex tended to form synaptic connection with the Cr+ neurons in the ventral horn, to indirectly activate the spinal motor neurons and control the fine activities of individual limbs. In addition, at the EM level, only 16.51 ± 5.75% of the BDA+ fibers targeted the dendrites of Cr+ neurons, and 65.38 ± 5.03% of the BDA+ fibers targeted the dendrites of unmarked neurons, while there was no significant difference in the size of these positive fibers. This finding reveals that the projection of CST on the spinal gray matter also makes synaptic contact with other neurons, such as other CBPs and choline acetyltransferase, which play an activity-dependent role in nutrition and affect the development and growth of these neurons, participating in sensorimotor integration in a complex manner by controlling the subsystems of different spinal circuits (Han et al., 2013; Jiang et al., 2018; Ueno et al., 2018). Moreover, we observed that the dendrites of Cr+ neurons received not only CS projections but also a large number of unmarked terminals. The number of these BDA- terminals was far greater than that of BDA+ terminals, and the size of these two terminals was significantly different. In addition to nociceptive and innocuous inputs, Cr+ neurons located in the dorsal horn can be connected with the parabrachial tract of the spinal cord to affect its activation (Petitjean et al., 2019). Other studies have found that rats can walk on open ground even without CST input, demonstrating that there are other descending pathways that can control the spinal motor neural network (Kanagal and Muir, 2009; May et al., 2017). In addition, the unilateral injection of tracer and electrophysiological evidence revealed that the vestibulospinal and reticulospinal tracts could form connections with spinal cord interneurons, indirectly affecting spinal cord motor neurons and regulating movement (Cabaj et al., 2006; Williams et al., 2010; Brockett et al., 2013). Combined with the results of this study, Cr+ interneurons may form a complex local circuit with primary afferent fibers, descending projecting neurons from the superior center or other interneurons, and participate in sensory afferents and efferents to regulate the activity of motor neurons in the spinal cord in addition to the regulation of CS descending fibers.

In conclusion, the morphological characteristics, distribution and synaptic connections of CS projections and Cr+ neurons in the dorsal and ventral horn of the cervical spinal cord gray matter of the experimental rats were observed and compared using immunohistochemistry and immunoelectron microscopy. There are some differences in the function of the dorsal horn and ventral horn of the spinal cord. The distribution characteristics and morphological structure of CS projections and Cr+ neurons in different regions of the gray matter indicate that they have different functions in the spinal cord. Moreover, Cr+ neurons can form direct synaptic connections with CS projections from the M1 area and tend to form ventrally, indicating that CS neurons projected from the M1 area innervate Cr+ neurons in the spinal gray matter, indirectly activating spinal motor neurons to regulate motor activity.

## Data availability statement

The original contributions presented in this study are included in the article/supplementary material, further inquiries can be directed to the corresponding authors.

## Ethics statement

This animal study was reviewed and approved by the Animal Care and Use Committee of Sun Yat-sen University.

## Author contributions

WL, SC, ZH, and XZ involved in the design and planning of the study. ZH and LS performed the tri-labeling and double-labeling experiments for LM and EM. ZH, XZ, and YZu performed the animal experiments and neural tracking. TC and ZC performed the immunohistochemical assay. ZH, LJ, and LO contributed to the single-labeling experiments for LM and EM. ZH involved in analysis of the dataset. WL, SC, XZ, and LO contributed to the study resources. All authors contributed to the article and approved the submitted version.
